# Perspective from Ecuador, the Second Country with More Confirmed Cases of Coronavirus Disease 2019 in South America: A Review

**DOI:** 10.7759/cureus.7452

**Published:** 2020-03-28

**Authors:** Alejandro Hallo, Alejandra Rojas, Carlos Hallo

**Affiliations:** 1 Internal Medicine, Hospital de Especialidad Eugenio Espejo, Quito, ECU; 2 Investigacion, Universidad Central del Ecuador, Quito, ECU; 3 Internal Medicine, New York University (NYU) Langone Health, Manhattan, USA

**Keywords:** sars-cov-2, covid-19, ecuador

## Abstract

To face the pandemic outbreak of a novel coronavirus many countries developed a series of containment methods; however, developing countries in South America had reacted apathetically to this worldwide concern. Ecuador's response to the novel virus Coronavirus Disease 2019 (COVID-19) started on February 26, 2020, one month after the outbreak began in China. As of today, the countries with more confirmed cases in South America are Brazil and Ecuador. Although Brazil has two times the number of cases than Ecuador (Brazil: 700 cases per 100000 people vs. Ecuador: 400 cases per 100000), the huge population difference between the two countries raises concerns about the public health policies implemented by the Ecuadorian government. Even though there is no cure for COVID-19, chloroquine and hydroxychloroquine are promising alternatives. The COVID-19 pandemic outbreak has shown that there is room for improvement in the healthcare systems worldwide and the disastrous results on the fragile often unprepared are those systems in developing countries.

## Introduction and background

Alarm bells over the world started to ring after the news regarding a case of pneumonia caused by a novel virus in Wuhan, China. The virus supposedly originated in one of the largest markets in Wuhan. On January 7, 2020, the Centers for Disease Control and Prevention (CDC) isolated the virus and called it severe acute respiratory syndrome coronavirus 2 (SARS-CoV-2). The disease caused by this virus was named Coronavirus Disease 2019 (COVID-19) by the World Health Organization (WHO).

Even though the majority of cases present with flu-like symptoms, 3.4% of patients develop severe pneumonia, septic shock, respiratory failure, and multiorgan failure leading to a fatal outcome [1-2].

On January 30, 2020, the WHO declared the COVID-19 outbreak a global health emergency. Countries applied many measures, hoping to contain the spread of the disease; for example, restricting mobility and quarantines. Additionally, incoming flights from countries with a high prevalence of the disease were suspended, first from China and then followed by Italy and Spain.

In South America, Brazil is the country with the highest number of COVID-19-confirmed cases followed by Ecuador. Although Brazil has two times the number of cases than Ecuador, it is important to analyze the prevalence of the disease in the context of the countries’ population. While Brazil, with a population of 212,559,417, has a total of 1,549 confirmed cases (0.007%); Ecuador, with a population of 17,567,751, has 789 confirmed cases (0.004%) [3-4]. The greater ratio of cases per population in Ecuador raises the concern about the efficacy of the containment measures and public health policies taken by the country.

The limitations of the healthcare systems in developing countries increase their chances of being affected more during a pandemic outbreak.

## Review

Confirmed cases and Ecuadorian response

After the announcement that COVID-19 was spreading rapidly across Asian countries, European countries went into an alarm state and started to design containment measures while South American countries reacted apathetically, delaying decisions on preventive measures, underestimating the severity of the events.

Ecuador's response to the novel virus COVID-19 started on February 26, 2020. The set of measures included monitoring the temperature and other COVID-19-related symptoms in travelers from high-risk countries [5]. On February 29, 2020, Ecuador confirmed the first case of COVID-19. The patient had arrived from Spain two weeks before the diagnosis was made [6].

After the WHO declared COVID-19 as a pandemic outbreak on March 11, 2020, Ecuador declared a national health emergency [7-8]. All non-essential activities were suspended indefinitely and the use of virtual platforms was encouraged for academic activities, jobs, and medical care. As did other countries did, Ecuador also closed its land, air, and sea borders to citizens and non-citizens for 21 days [7,9]. Restrictions to vehicular or pedestrian traffic and a national curfew from 7 pm to 5 am also took place [9-10].

Despite these measures, the virus continued spreading across the Ecuadorian provinces. On March 23, 2020, 789 confirmed patients with COVID-19 and 18 deaths related to the virus were reported by the Ministry of Health of Ecuador [11]. The mortality rate was similar to other countries [10,12]. The number of confirmed patients increased, so did the number of people in epidemiological monitoring and quarantine.

The infection risk in physicians also increases in countries with a high prevalence of SARS-CoV-2. In Italy, more than 3,000 health providers were infected becoming an increasing concern among health providers worldwide. In Ecuador, 24 doctors have been tested positive for the virus one month after the first patient's admission with COVID-19. The rising number of physicians testing positive for COVID-19 raises concern amid a growing outcry over the shortage of protective equipment and medical supplies [13].

Guayas, with 607 confirmed cases, is by far the most affected province by COVID-19 in Ecuador. Measures applied in the rest of the country with relative success failed in this province. The out-of-control increase in confirmed cases poses the risk of collapsing the health care system there. This situation led the government to declare the province as a national security zone, leaving operational planning to the army [8,14].

Transmission

The transmission was described as animal-to-person in affected patients at the Huanan Seafood Wholesale Market of Wuhan [15]. At the moment, person-to-person transmission and fomite transmission have been linked with the spread of the illness [16]. Li compiled information on the dynamics of the novel virus, estimating a transmission rate of R0 2.2 for COVID-19 [7]. In addition, Cascella mentions that close contact with an infected person is needed to transmit the virus. Therefore, relatives and health professionals are at greater risk [15].

There are some discrepancies about the transmission. While some studies support the animal-to-human transmission, including a case of a pet that tested positive for COVID-19, the CDC states that there is no evidence that pets can transmit the virus or present the symptomology of infection [17-19].

Prevention

The main goal in the control of a pandemic outbreak is to achieve R0 less than 1 (one positive patient can infect less than one extra person). To reach this goal, prevention is paramount [15].

The WHO and CDC recommend avoiding contact with symptomatic people and agglomerations [20-22]. Basic hygiene practices, such as constant hand washing and the use of face masks in symptomatic patients, are also recommended to prevent the spread of the virus [20].

Many countries utilized quarantine and mobilization restrictions to control the spread; however, these measures may not be sufficient in developing countries such as Ecuador [23].

Pathophysiology

The cause of COVID-19 is a ribonucleic acid (RNA) virus of the Coronaviridae family called SARS-CoV-2. Although the exact pathophysiology of SARS-CoV-2 is unknown, its structural similarity with other viruses of the Coronaviridae family allows an understanding of its dynamics and the evolution of the disease [16]. The clinical presentation may vary from flu-like symptoms to severe pneumonia similar to SARS-CoV and Middle East respiratory syndrome-related coronavirus (MERS-CoV) [24].

Channappanavar and Granlinski mention that the most aggressive strains of the Coronavirinae family, like SARS-CoV, have protein E in their envelope that stimulates NF-kB, a protein complex involved in cellular responses, including stress, cytokine signaling, response to viral infections, and apoptosis. Protein E, along with other proteins, interferes with the host immune response (tumor necrosis factor (TNF) alpha and interleukin 6 (IL-6)) [24]. These processes produce uncontrolled inflammation, resulting in lung edema and fibrosis [24-25]. 

Treatment

Efforts to control COVID-19 had been made by many countries, starting with investigations to develop a vaccine from the attenuated or inactive virus [26]. The National Institute of Allergy and Infectious Diseases announced the beginning of the first trial on 45 volunteers between 18 and 55 years old. This first phase is estimated to last for six weeks [27]. The Kaiser Research Institute is evaluating the feasibility of the mRNA-1723 vaccine [28].

The management of patients with acute respiratory syndrome consists of assisted oxygen ventilation to achieve a saturation percentage of oxygen (SpO2) of 90% and antipyretics [16].

Multiple drugs are being studied to treat COVID-19, with promising results. In the United States and China, a randomized clinical trial has been initiated to test the efficacy of remdesivir [29]. At the moment, oseltamivir, a neuraminidase inhibitor, has been used more extensively [16].

Chloroquine, a drug used worldwide, has a promising effect in the treatment of severe pneumonia by COVID-19 at a dose of 500 mg^2 ^times a day [30-31]; however, Zhou questions its use due to its side effects. Instead of using chloroquine, Zhou suggests hydroxychloroquine as an alternative treatment [32]. Hydroxychloroquine seems to attenuate the progression to more severe scenarios of COVD-19 infection, although its dosage still needs further study [31].

## Conclusions

The low mortality of COVID-19 may be the reason for developing countries underestimating the magnitude of this pandemic outbreak. The health care systems risk collapsing due to the increasing number of confirmed patients who need intensive care and the spread of the disease in health providers. The high demand for financial and human resources by this disease leads to a collateral increase in mortality from other causes. The COVID-19 pandemic outbreak has shown that there is still room for improvement in healthcare systems worldwide to prevent its catastrophic impact, especially on the fragile and often unprepared systems in developing countries.
